# New Remedies and Their Application

**Published:** 1893-07

**Authors:** L. P. Bethel

**Affiliations:** Kent, O.


### New Remedies and their Application.

BY L. P. BETHEL, M.D., D.D.S., KENT, O.

Read before the Northern Ohio Dental Association, at Akron, May, 1893.

Of the many new drugs recently put on the market several are applicable in dental practice; as the following:

PYROZONE.* — This drug is prepared in three different strengths. Medicinal pyrozone is a preparation containing three per cent. of hydrogen peroxide, combined in such a way as to render the solution stable. Peroxide of hydrogen as ordinarily prepared contains, when fresh, fifteen volumes of the confined gas and is in reality a three-per cent. solution. Peroxide of hydrogen deteriorates quite rapidly at the ordinary temperature, as gas is slowly given off at a temperature of 34° F. and increases in rapidity as the temperature is raised. Medicinal pyrozone deteriorates very slowly, if at all. In experimenting I placed a quantity in a wide-mouthed bottle and left uncorked, exposed to light and air, in a warm room, while the original bottle was placed in a dark, cool place. At the end of ten days the two were tested in pockets around the roots of teeth, congested and spongy gums, and on pure pus cultures, with apparent indistinguishable strong effervescence in each.

Medicinal pyrozone has no deleterious action on the tissues of the mouth, is non-poisonous and unirritating. It may be used with good effect for cleansing root canals, as an injection about the necks of teeth after the removal of calculus, for the removal of green stain from the teeth, as a cleanser of pus cavities, and in fact wherever peroxide of hydrogen is advocated. It is also a hemostatic and a bleacher of tooth substance.

Antiseptic pyrozone is a five-per cent. solution, and the caustic pyrozone, a twenty-five-per cent. solution. These are ethereal solutions, inflammable and volatile. They should be kept in a dark, cool place, well corked. Upon evaporation of the ether, however, the antiseptic solution gets stronger, instead

*Manufactured by McKesson & Robins, New York City.

of weaker, and may become caustic. Applied to the skin or mucous membrane the antiseptic pyrozone causes a burning and prickling sensation and leaves a white stain, which is more of the nature of a bleached spot than a true eschar. It disappears after one to three hours, and the tissues return to apparently a normal condition. The caustic pyrozone acts about the same with possibly more of a burning sensation after the application. Both of these preparations should be placed where wanted, and it is best to use but a limited quantity of the medicament, so that it will not spread out over healthy tissues. The tissues may be protected, however, by smearing the parts with glycerine. Glycerine or glycerite of tannin will relieve the smarting and tingling of the pyrozone. The pyrozone preparations act more energetically on moist than on dry surfaces. The antiseptic pyrozone is used in root canals for the removal of green stain, to stop suppuration and reduce inflammation from whatever cause, as an application in pyorrhea alveolaris, etc. The caustic preparation is used principally for the treatment of pyorrhea alveolaris. The addition of trichloracetic acid seems to make it even more effective. This acid being a solvent of calculus removes all remaining incrustations from scaling, and it is a stimulating astringent. The pyrozone preparations are all bleachers and hemostatics. They are all non-coagulators of egg albumen. In the ethereal solutions, aside from the destructive action of the gas on micro-organisms, the ether in itself is a good antiseptic and will kill most bacteria. It should be borne in mind that pyrozone causes effervescence, not only in the presence of pus, but of blood, and in fact wherever there is foreign organic matter. The effervescence is, therefore, not a sure indication of the presence of pus.

In these three preparations we have a wide range of useful drugs.

Eucalyptus and Thymol Antiseptic.*—This is a preparation containing borate of soda, benzoic acid, boric acid, thymol, oil of eucalyptus, oil of wintergreen, oil of thyme, oil of peppermint, and fl. extract of wild indigo. It may be used externally or internally. It is a non-coagulator of egg albumen. It may

*Manufactured by Parke Davis & Co., Detroit, Mich.

be freely used as a mouth wash, root dressing, general detergent, for cleansing the hands, and wherever a good antiseptic is desired. It may be used freely about the mouth as it is non-poisonous, non-irritating and not injurious to tooth substance.

ALUMNOL.*—An Aluminum salt. It is a white non-hygroscopic powder readily soluble in water, and suitable for application in various forms. Solutions may be from one per cent. to ten per cent. It arrests suppuration and secretion, and hastens the closing of wounds. Its use is indicated in the irrigation of cavities, abscesses, infected wounds, as an application in pyorrhea pockets, etc. It is an antiseptic, astringent, a strong coagulator of egg albumen, and possesses hemostatic properties.

ACETO TARTRATE OF ALUMINUM. — This is another salt of aluminum possessing antiseptic, astringent and hemostatic properties. It is a strong coagulator of egg albumen. It is useful for controlling hemorrhage after extraction of teeth, irrigating cavities, abscesses, infected wounds, as an application to congested gums, pyorrhea pockets, etc.

JOHNSTON'S ETHEREAL ANTISEPTIC SOAP.† — This is one of the nicest preparations for rendering the hands antiseptically clean that I have seen. It is in the form of a liquid soap. When used it has a strong ether odor, but no odor remains after rinsing the hands, and they are left white with a soft velvety feeling to the skin. Bichloride of mercury may be added to the soap without precipitation, an advantage over other forms of soap.

With a view of testing the comparative values of the above antiseptics, pure cultures of pyocyneus (a bacillus found in pus) were used by taking a small quantity of the antiseptic and placing on a glass slide, mixing in some of the culture, and placing a cover glass over this, examining through the microscope. This bacillus is actively motile, and when movement was suspended it indicated that the germs were killed. Caustic, Antiseptic and Medicinal pyrozone acted immediately ; no movement was seen after the slides were examined under the microscope. Medicinal pyrozone that had been exposed to light and air in a warm room

†Lahn & Fink, New York. Importers.
*Manufactured by Parke Davis & Co., Detroit, Mich.

for ten days caused suspension of movement after one minute. Johnston's ethereal antiseptic soap acted immediately; no motion observed. Alumnol, ten per cent. solution, no movement after one minute. Eucalyptus and thymol antiseptic solution, no movement after four minutes. Aluminum aceto tartrate, five per cent. solution, no movement after five minutes.

Some allowance must be made, however, in this test, for varying quantities of microbes and antiseptic solutions. An antiseptic must permeate the albuminous coating of the micro-organism before it can kill; in some of the cultures the micro-organisms were massed together more closely than in others, and hence would take longer time for the antiseptic to act on these, although there seemed to be comparatively little difference in suspension of movement in the individual and massed microbes.

Other tests were made on pure cultures of pus cocci, the staphylococcus pyogenes aureus, one of the most resistent of the pus microbes. This micro-organism is not motile, hence cultures were made in gelatine tubes and a small amount of the antiseptic placed immediately over the culture. This was left but a moment and the tubes set aside. If the culture developed after several days it indicated that the micro-organisms were not all killed. The result of this experiment was that the micro-organisms began developing on the fifth day after the application of the pyrozones, on the seventh day after the application of the antiseptic soap, on the second day after the application of aceto tartrate of aluminum and alumnol, in one and a half days after the application of eucalyptus and thymol solution.

This experiment showed that these drugs have a growth hindering effect, but do not kill resistant bacteria on short contact.

Further experiments on this same bacteria were made by leaving the various solutions in contact with the cultures for five minutes. The result of this was that after twelve days no growth of the micro-organisms took place. This showed that all of these preparations are good antiseptics if left in contact with germs for a few minutes. Further experiments, as a mixed culture of bacteria taken from a root canal, showed no growth after these drugs had been left in contact with the cultures for five minutes.

As an efficient tonic preparation for patients, Weld's Syrup of Iron Chloride* seems to be the best. It is compounded in such a way that it has no injurious effect upon the teeth that the ordinary preparations of iron have.

Coca Cordial* is also highly recommended as an efficient and pleasant tonic. It is often advisable for the dentist to prescribe a tonic both prior to and after an operation, and he can do no better than to recommend the above.

Sodium Peroxide has been mentioned as a useful drug in dentistry. Its action is similar to peroxide of hydrogen, and as a bleacher of discolored teeth it is highly recommended. I have as yet made no experiments with this preparation.

Tropa Cocaine is the newest local anæsthetic. It is said to be as efficient as cocaine, but only half as poisonous. Its present cost—$240 an ounce, however, practically places it beyond the average dentist.

